# Target-Oriented Screening of Traditional Chinese Medicine Potential Inhibitors Against African Swine Fever Virus dUTPase and Preliminary Evaluation of Enzymatic Activity Effects

**DOI:** 10.3390/v18080809

**Published:** 2026-07-23

**Authors:** Mengqi Zhao, Xinyu Dai, Hao Tang, Changde Wu, Junjuan Fan, Shouhua Feng, Kang Ou, Gen Lu, Xuesong Fang, Xiaoyue Chen, Shu Wei, Zuofeng Yang, Jinling Liu

**Affiliations:** 1 Key Laboratory of Livestock Infectious Diseases, Ministry of Education, and Key Laboratory of Ruminant Infectious Disease Prevention and Control (East), Ministry of Agriculture and Rural Affairs, College of Animal Science and Veterinary Medicine, Shenyang Agricultural University, 120 Dongling Road, Shenyang 110866, China; zhaomengqicau@163.com (M.Z.); daixin31415926@163.com (X.D.); 13889639416@163.com (H.T.); wuchangde@syau.edu.cn (C.W.); kyxtc_007@163.com (K.O.); genlu_cn@163.com (G.L.); 2 College of Animal Husbandry and Veterinary Medicine, Liaoning Vocational College of Agriculture and Technology, No. 76-0 Yucaili, Bayuquan District, Yingkou 115009, China; junjuanfan@163.com; 3 Wens Farming and Animal Husbandry Co., Ltd., Liucheng Subdistrict, Chaoyang County, Chaoyang 122000, China; shouhuafeng123@163.com; 4 Liaoning Yaho Nutrition Technology Co., Ltd.—Animal Husbandry Standard Feed Branch, No. 16, Haiqian Road, Xingcheng Coastal Economic Zone, Huludao 125100, China; fangxuesong@hotmail.com; 5 The Preventive and Control Center of Animal Disease of Liaoning Province, Liaoning Agricultural Development Service Center, No. 95, Renhe Road, Shenbei District, Shenyang 110164, China

**Keywords:** African swine fever virus (ASFV), Traditional Chinese Medicine (TCM) inhibitors, virtual screening, ASFV dUTPase, molecular dynamics simulation

## Abstract

African swine fever (ASF), caused by African swine fever virus (ASFV), is a highly contagious disease that has brought severe economic losses to the global swine industry. ASFV dUTPase is a key enzyme modulating viral nucleotide metabolism and genome stability, serving as a specific structural target for anti-ASF lead discovery. Based on the crystal structure of ASFV dUTPase (Georgia 2008/1 strain), we established a molecular docking model to virtually screen a Traditional Chinese Medicine (TCM) compound library following ADME-based filtration. We further conducted 100 ns molecular dynamics (MD) simulations via GROMACS and calculated binding free energies using the MM/PBSA method, followed by in vitro enzymatic inhibition assays for verification. Four candidate compounds, namely Salvianolic acid B, Kukoamine B, Ligustroflavone and 9‴-Methyl salvianolic acid B, showed favorable structural accommodation and binding potential. In vitro tests indicated that Ligustroflavone and 9‴-Methyl salvianolic acid B exhibited preliminary inhibitory activity against ASFV dUTPase, with IC_50_ values of 1.87 mM and 0.92 mM, respectively, while Kukoamine B showed no inhibitory effect. This study identified potential TCM-derived lead molecules targeting ASFV dUTPase, providing new references and candidate compounds for the development of anti-ASF therapeutics.

## 1. Introduction

African swine fever (ASF) is a highly contagious, febrile, and lethal infectious disease of swine caused by the African swine fever virus (ASFV). Despite its persistent threat to the global swine industry, no safe and effective commercial vaccine or specific antiviral therapeutic is currently available [1]. This necessitates the development of novel antiviral strategies, among which targeting the key enzymes responsible for viral genomic replication has emerged as a compelling research direction.

Deoxyuridine triphosphatase (dUTPase; EC 3.6.1.23) is a critical enzyme for maintaining the fidelity of genomic replication. It catalyzes the hydrolysis of deoxyuridine triphosphate (dUTP), thereby maintaining a low intracellular ratio of dUTP to dTTP and preventing the misincorporation of uracil into DNA [2]. As a large, double-stranded DNA virus, ASFV encodes its own dUTPase (ASFV dUTPase) [3,4]. Previous studies have demonstrated that this non-structural protein is essential for viral replication, as it protects the viral genome from damage by hydrolyzing dUTP during DNA synthesis [4]. Crucially, ASFV predominantly infects resting porcine macrophages, which exhibit extremely low levels of endogenous dUTPase. Therefore, the virus is obligately dependent on its self-encoded dUTPase for efficient replication [4]. This distinct host-virus disparity provides a robust molecular foundation for enabling the design of highly selective antiviral drugs with minimal cytotoxicity. However, recent functional genomics studies have revealed that the essentiality of this enzyme is distinctly strain-dependent [5]. Specifically, while deletion of the dUTPase-encoding E165R gene in attenuated or tissue-adapted strains (such as BA71V) typically leads to impaired viral replication to varying degrees in primary porcine macrophages [4,5], the same genetic deletion in highly pathogenic virulent isolates (such as the Georgia 2008/1 strain) does not induce significant alterations in either in vitro replication kinetics or in vivo pathogenicity [5,6]. This phenotypic divergence indicates that alternative metabolic bypasses or host-derived compensatory mechanisms might facilitate viral survival in certain isolates despite the loss of viral-encoded dUTPase. Therefore, objectively defining the biological boundaries of this target and conducting rational lead discovery based on its highly conserved crystalline conformation holds substantial reference value for enriching the chemical space of potential anti-ASFV small-molecule scaffolds.

Despite this strain-dependent variation, the catalytic core domain of ASFV dUTPase remains highly conserved in both sequence and conformation across sequenced isolates [5,7], and its essential role in viral replication has been validated under specific genetic contexts [4]. Structurally, ASFV dUTPase differs markedly from the host swine enzyme. The host enzyme adopts a classical three-subunit active-site architecture, whereas the ASFV enzyme forms a novel two-subunit configuration, driven by a unique orientation of its C-terminal β-strand after motif IV [3]. In this viral architecture, each catalytic cleft is independently gated by the folded-back C-terminal arm. This profound conformational divergence from the host counterpart not only creates a virus-specific binding interface [3], but also offers a distinct advantage for the rational discovery of selective small-molecule inhibitors targeting the conserved active pocket. Unlike traditional strategies that directly inhibit viral DNA polymerases, dUTPase inhibition disrupts the intrapopulation dUTP/dTTP metabolic equilibrium, leading to massive uracil misincorporation into the viral genome and ultimately triggering “lethal mutagenesis” [2]. By compromising the integrity of genetic information to suppress viral replication, this mechanism theoretically increases the barrier to resistance, reducing the risk of viral escape through single-point mutations. However, research into small-molecule inhibitors that specifically target ASFV dUTPase is still in its infancy, falling behind advances in inhibitors against mammalian host dUTPase, including the oncology clinical candidate TAS-114 and various nucleoside derivatives [8]. This gap mainly stems from intrinsic bottlenecks restricting current anti-ASFV drug development. To date, no dedicated antiviral drugs have been licensed for veterinary use, and conventional live-virus screening strictly requires BSL-3 containment laboratories. Such facility constraints inevitably restrict large-scale research deployment and extend experimental timelines [9]. Accordingly, computational screening performed under standard laboratory conditions to discover natural product-derived scaffolds with favorable safety profiles offers a feasible and safe alternative to prioritize lead candidates.

Given the significant potential of ASFV dUTPase as an antiviral target, specific small-molecule inhibitors remain sparsely reported. To facilitate lead discovery while reducing empirical trial-and-error costs, Computer-Aided Drug Design (CADD) has been widely applied to identify small-molecule leads targeting critical viral non-structural proteins [10,11]. Notably, integrating CADD with TCM compound libraries has been shown to be useful in screening candidate compounds [11,12,13]. TCM libraries are known for their structural diversity, broad biological activities, and favorable safety profiles [14], making them a valuable resource for developing veterinary antiviral therapeutics.

In this study, we integrated hierarchical virtual screening, molecular dynamics (MD) simulations, and in vitro enzymatic assays to identify potential ASFV dUTPase inhibitors. Utilizing the crystal structure of ASFV dUTPase [3], candidate compounds were initially screened from a TCM database via molecular docking. To account for receptor flexibility and evaluate binding thermodynamics under near-physiological conditions, the candidate complexes were further subjected to all-atom MD simulations and MM/PBSA-based binding free energy calculations [15]. Finally, the inhibitory activities of the top-ranked compounds were validated experimentally. This workflow provides candidate lead compounds derived from TCM and a structural basis for future anti-ASFV drug design.

## 2. Materials and Methods

### 2.1. Construction of the TCM Compound Library

The three-dimensional (3D) structures of small molecules derived from natural herbs were retrieved from the Traditional Chinese Medicine Systems Pharmacology Database and Analysis Platform (TCMSP, https://tcmsp-e.com/tcmsp.php, accessed on 10 January 2026) [16]. Pharmacokinetic pre-filtering based on an oral bioavailability (OB) threshold of ≥30% and a drug-likeness (DL) threshold of ≥0.18 was applied prior to molecular docking. Ligand preparation was performed using the prepare_ligand.py script from MGLTools (v1.5.7). All compounds were converted to the PDBQT format, with the addition of Gasteiger charges and the assignment of rotatable bonds. The -U lps parameter was applied to preserve non-polar hydrogen information in the output files.

### 2.2. Target Protein Pre-Processing

The crystal structure of ASFV dUTPase (PDB ID: 6KY9, resolution: 1.70 Å) was obtained from the RCSB Protein Data Bank. The target protein sequence corresponds to the UniProt accession number A0A2X0SE53, derived from the African swine fever virus Genotype II strain Georgia 2008/1. For the homotrimeric coordinates utilized in both docking and molecular dynamics simulations, Chains A, B, and C all encompass residues 2–149. PyMOL (v3.0.0, Open-Source) [17] was utilized to remove crystallographic water molecules, co-crystallized ligands, and metal ions. Following hydrogen atom addition and initial energy minimization, the protein structure was refined using the 3Drefine server [18]. The overall structural quality was assessed using the RamachanDraw package in Python 3. Given that the highly flexible C-terminal arm segment (residues 151–165) of the 6KY9 crystallographic structure was unresolved in the crystallographic coordinates, the virtual screening and initial molecular dynamics simulations focused on the ordered catalytic core domain within this structure. Finally, AutoDock Tools (v1.5.7) [19,20] was employed for protonation, charge assignment, and conversion to the PDBQT format.

### 2.3. Virtual Screening of the TCM Database

Virtual screening of the TCM compound library against ASFV dUTPase was performed using the WatVina program, which is developed based on the AutoDock Vina [21] architecture. Based on literature reports [3], the cleft formed by residues Arg27, Arg71, and Ser72 of Chain A, as well as Asp91 and Tyr94 of Chain B, was defined as the active site. The GetBox plugin (v1.1) within PyMOL was employed to determine the docking box coordinates centered on the active pocket. Specifically, the center of the grid box was anchored at x = 17.8, y = −19.0, and z = 20.1, with the box dimensions configured to x = 27.6, y = 22.1, and z = 23.3 Å to encompass all critical catalytic residues (Arg27, Arg71, Ser72, Asp91, and Tyr94). The global search exhaustiveness parameter was configured to 32, and the maximum number of binding modes to generate per ligand was set to 9. Pharmacophore geometric constraints were enabled, with all other parameters maintained at WatVina defaults. Upon completion, ligand conformations were ranked based on their docking scores, applying a stringent screening threshold of −8.0 kcal/mol to prioritize top candidates [22]. The compounds were named using their common chemical names or Chinese names. To validate the reliability of the molecular docking protocol, a redocking procedure was conducted by extracting the co-crystallized dUMP ligand from the structure and re-introducing it into the catalytic pocket. The docking algorithm successfully sampled the experimental crystallographic binding mode. Among the top-ranked binding poses, we identified a conformation that highly reproduced the native geometry, yielding a heavy-atom Root Mean Square Deviation (RMSD) of 1.269 Å. This quantitative alignment verifies the fidelity and target-adaptability of our defined grid box and screening setup. Finally, the interaction patterns between the candidate compounds and the active site of ASFV dUTPase were analyzed using the PLIP (Protein-Ligand Interaction Profiler) online tool [23].

### 2.4. Molecular Dynamics (MD) Simulations

MD simulations (100 ns) were performed for the unliganded 6KY9 protein and the four protein-ligand complexes using GROMACS 2022.3 [24]. Ligands were parameterized with the GAFF force field and AM1-BCC charges using Antechamber (AMBER) and Acpype to generate the corresponding topology files compatible with GROMACS [25,26]. The Amber14sb force field was applied to describe the protein. Each system was centered in a cubic box with a minimum distance of 1.0 nm to the boundaries under periodic boundary conditions. Solvation was performed using the TIP3P water model [27], and 0.15 M NaCl was added to neutralize the system. Energy minimization was conducted in two stages: steepest descent (50,000 steps; force threshold: 5.0 kJ/mol) followed by conjugate gradient (50,000 steps; force threshold: 2.0 kJ/mol). Long-range electrostatic interactions were treated using the Particle-Mesh-Ewald (PME) method [26], with short-range van der Waals and electrostatic cutoffs set to 14 Å. Bond lengths involving heavy atoms were constrained using the LINCS algorithm.

The systems were equilibrated for 300 ps in the NVT ensemble (300 K, Parrinello-Donadio-Bussi thermostat) [28], followed by 300 ps in the NPT ensemble (1 atm, Parrinello-Rahman barostat) [29]. Finally, a 100 ns production run was performed at 300 K and 1 atm with a 2 fs time step.

Trajectory analysis included Root Mean Square Deviation (RMSD), Root Mean Square Fluctuation (RMSF), Radius of Gyration (Rg), Solvent Accessible Surface Area (SASA), and hydrogen bond counts. Free Energy Landscapes (FEL) were generated based on the first two principal components (PC1 and PC2). Visualization was performed using PyMOL [17], ChimeraX (v1.4) [15], and Origin 2022 [30].

### 2.5. Molecular Mechanics Poisson–Boltzmann (Generalized Born) Surface Area [MM/PB(GB)SA] Calculations

The binding free energies (ΔG_bind_) between ASFV dUTPase and the four candidate compounds were calculated using MM/PBSA and MM/GBSA methods implemented in the gmx_MMPBSA toolkit. The binding free energy is expressed as

(1) $${\Delta G}_{\mathrm{bind}}=G_{complex}-(G_{protein}+G_{ligand})$$

where each free energy term includes molecular mechanics energies (van der Waals and electrostatic interactions), solvation free energies (polar and non-polar contributions), and configurational entropy. To ensure thermodynamic equilibrium, structural frames were extracted from the final 20 ns of the 100 ns MD trajectories for binding energy analysis.

### 2.6. Protein Expression and Purification

The *E. coli* BL21(DE3) strain harboring the pET28a-His-dUTPase plasmid was cultured in LB medium (50 µg/mL kanamycin) at 37 °C until an OD_600_ of ~0.6 was reached. Expression was induced with 0.1 mM IPTG at 16 °C for 16 h. Cells were harvested by centrifugation (4 °C), resuspended in PBS (pH 7.4), and lysed using a high-pressure homogenizer. The supernatant was collected after centrifugation (12,000× *g*, 30 min, 4 °C) and filtered through a 0.45 µm membrane. The filtrate was loaded onto a Ni-NTA affinity chromatography column. Target proteins were eluted using a gradient of imidazole (up to 500 mM) in PBS. Purity was assessed by SDS-PAGE, and protein concentration was determined using a BCA protein assay kit (Thermo Fisher Scientific, Waltham, MA, USA).

### 2.7. Enzymatic Inhibition Assay

The catalytic activity of dUTPase was measured by the colorimetric reaction between ammonium molybdate and pyrophosphate (PPi), as described by Grindey et al. [31]. A PPi standard curve was established to correlate OD_575_ with concentration (µM). The 100 µL reaction mixture contained 50 mM Tris-HCl (pH 8.0), 5 mM MgCl_2_, 1 mM DTT, 0.2 mM dUTP, and approximately 4.4 µg of purified dUTPase. The candidate compounds were initially dissolved in 100% DMSO to prepare concentrated stock solutions and subsequently diluted with the assay buffer to generate working stock solutions at concentrations of 0.01, 0.1, and 1.0 mM. For each assay, 1 µL of the respective working stock solution was added to the 100 µL reaction mixture, yielding final working concentrations of 0.1, 1, and 10 µM, respectively. The final concentration of DMSO in all groups was strictly maintained below 1% (*v*/*v*). A vehicle negative control (equal volume of DMSO) and compound-only blank controls (containing identical compound gradients in the reaction buffer without enzyme or substrate) were set up in parallel to correct potential spectrophotometric background interference originating from the intrinsic chromophoric scaffolds of natural products. After a 10 min pre-incubation at 37 °C, the reaction was initiated by adding dUTP, followed by 15 min of incubation. The reaction was quenched with ammonium molybdate-sulfuric acid solution, and OD_575_ was measured. Importantly, for samples yielding high colorimetric absorbances that exceeded the upper limit of the initial calibration curve (such as the inhibitor-free control group), a quantitative correction was implemented by introducing appropriate buffer dilution factors prior to measurement, or by using mathematical calibrations verified within an extended linear range, thereby ensuring that the analytical responses used for subsequent regressions strictly fell within the linear response domain governed by the Beer-Lambert law. Furthermore, to facilitate pharmacological interpretation and comparative analysis, the reporting unit for pyrophosphate yields throughout this manuscript was standardized to molar concentrations (μM). Each experiment was performed in triplicate. Raw optical density inputs for both the treatment groups and the negative vehicle controls were baseline-corrected by subtracting their corresponding compound-only blank values. The inhibition rate was calculated according to the following equation:

(2) $$Inhibitionrate(\%)=\left(1-\frac{{OD}_{575,sample}}{{OD}_{575,control}}\right)\times 100\%$$

where OD_575,sample_ represents the absorbance at 575 nm of the group with the inhibitor, and OD_575,control_ represents the absorbance of the control group (enzyme and substrate only, without inhibitor).

Statistical significance was analyzed using SPSS 22.0 (*t*-test or one-way ANOVA), and data were visualized with GraphPad Prism 8.0.

## 3. Results

### 3.1. Virtual Screening and Molecular Docking Analysis

**Figure 1:**
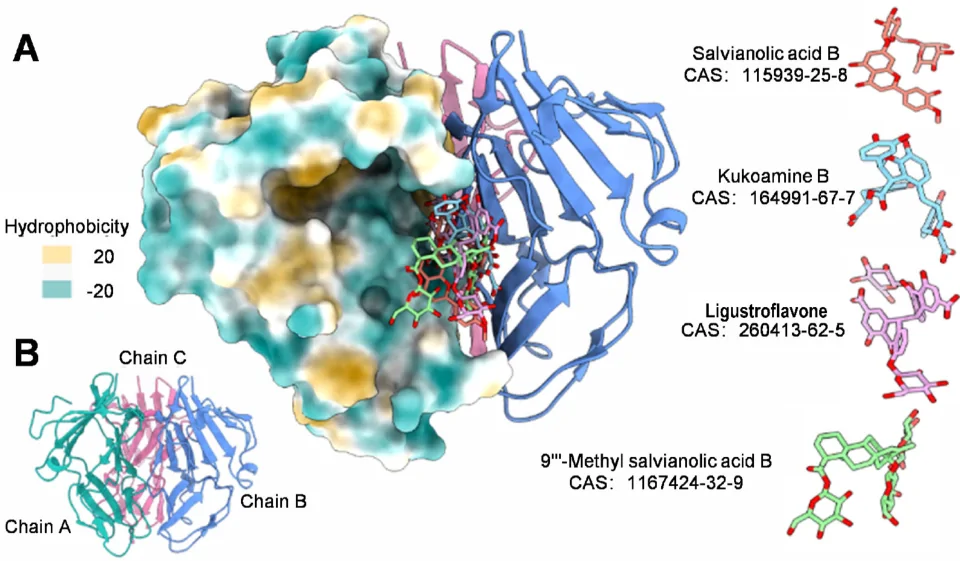
Molecular docking models of ASFV dUTPase with candidate inhibitors. (**A**) Surface hydrophilic and hydrophobic distribution of ASFV dUTPase. Cyan represents hydrophilic regions, and yellow represents hydrophobic regions. Four candidate small molecules are presented in stick representation. (**B**) Homotrimeric structure of ASFV dUTPase. Subunits A, B, and C are colored cyan, blue, and pink, respectively. The ligand-binding pockets at the subunit interfaces are clearly visible. The tested compounds include Salvianolic acid B, Kukoamine B, Ligustroflavone, and 9‴-Methyl salvianolic acid B.

Following the preliminary ADME-based pharmacokinetic filtration of the natural product database, a hierarchical virtual screening of the retained TCM compound library was conducted using a docking score threshold of ≤−8.0 kcal/mol. The top four compounds were identified as potential inhibitors: Salvianolic acid B (CAS: 115939-25-8), Kukoamine B (CAS: 164991-67-7), Ligustroflavone (CAS: 260413-62-5), and 9‴-Methyl salvianolic acid B (CAS: 1167424-32-9), with docking scores of −12.18, −12.02, −11.91, and −11.74 kcal/mol, respectively (Table 1). ASFV dUTPase functions as a homotrimer, characterized by three active sites located at the interfaces between adjacent subunits. Molecular docking predicted that all four candidates occupy the catalytic pocket formed by Chain A and Chain B (Figure 1). Nevertheless, given the inherent limitations of static scoring functions, which occasionally overlook entropic penalties and complex solvation effects, these initial docking scores were utilized primarily for early-stage scaffold enrichment rather than definitive potency ranking, thereby necessitating subsequent dynamic evaluations.

**Figure 2:**
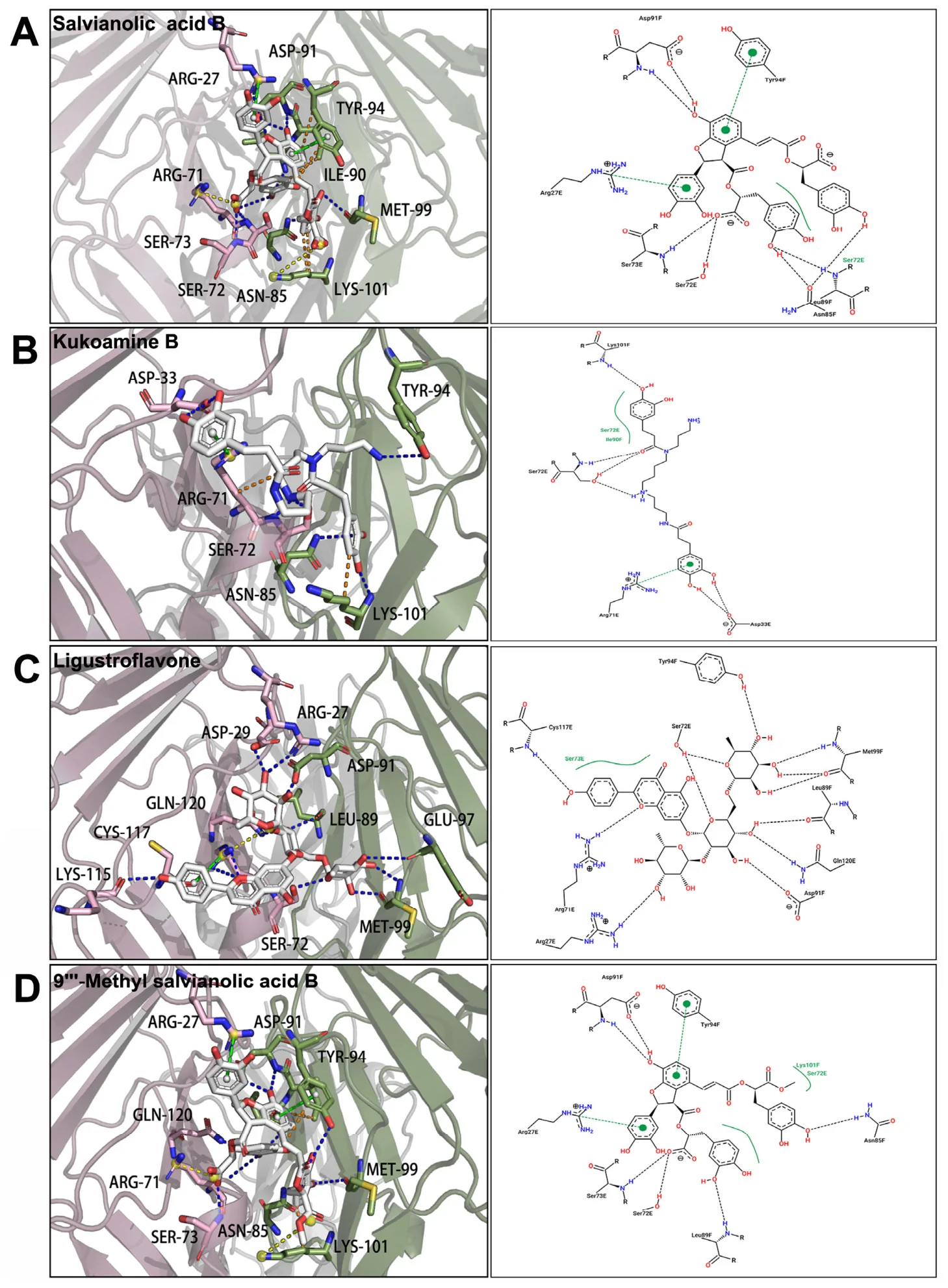
Molecular docking interaction patterns of four candidate inhibitors with ASFV dUTPase. (**A**) Salvianolic acid B, (**B**) Kukoamine B, (**C**) Ligustroflavone, and (**D**) 9‴-Methyl salvianolic acid B interacting with the binding pocket of ASFV dUTPase. The left panels show three-dimensional (3D) structural representations. The inhibitor molecules are displayed as stick models. Chain A and Chain B of the dUTPase protein are colored purple and green, respectively. Atoms are colored as follows: nitrogen in blue, oxygen in red, sulfur in yellow. Blue dashed lines denote hydrogen bonds, green dashed lines represent π-π stacking interactions, yellow dashed lines indicate polar interactions, and orange dashed lines represent hydrophobic interactions. The right panels show two-dimensional (2D) schematic diagrams illustrating the interaction patterns. Black dashed lines represent hydrogen bonds, and green solid lines indicate hydrophobic interactions.

An analysis of molecular interaction patterns (Figure 2) revealed that all four compounds established extensive hydrogen bond (H-bond) networks with residues in the active pocket. Among them, Salvianolic acid B and 9‴-Methyl salvianolic acid B exhibited the most dense interactions with key residues. Specifically, Salvianolic acid B formed H-bonds with Arg27, Arg71, Ser72, Ser73, and Asn85 of Chain A, as well as Asp91, Tyr94, Ile90, Met99, and Lys101 of Chain B (Figure 2A). Similarly, 9‴-Methyl salvianolic acid B interacted via H-bonds with Arg27, Arg71, Ser73, Gln120, and Asn85 of Chain A, and Asp91, Tyr94, Met99, and Lys101 of Chain B (Figure 2D). In addition to forming multiple H-bonds (Asp29, Gln120, Cys117, and Lys115 of Chain A; Arg27, Asp91, Leu89, Glu97, and Met99 of Chain B), the binding of Ligustroflavone was also stabilized by hydrophobic interactions mediated by Ser72 (Figure 2C). In contrast, Kukoamine B formed fewer H-bonds, involving only Asp33, Arg71, Ser72, and Asn85 of Chain A, alongside Tyr94 and Lys101 of Chain B (Figure 2B), suggesting a binding mode distinct from the other three candidates.

### 3.2. Molecular Dynamics (MD) Simulation Analysis

To evaluate the stability of the four candidate compounds in a simulated aqueous environment, 100 ns MD simulations were performed on the four dUTPase-inhibitor complexes and the apo-dUTPase.

#### 3.2.1. System Stability and Conformational Compactness

**Figure 3:**
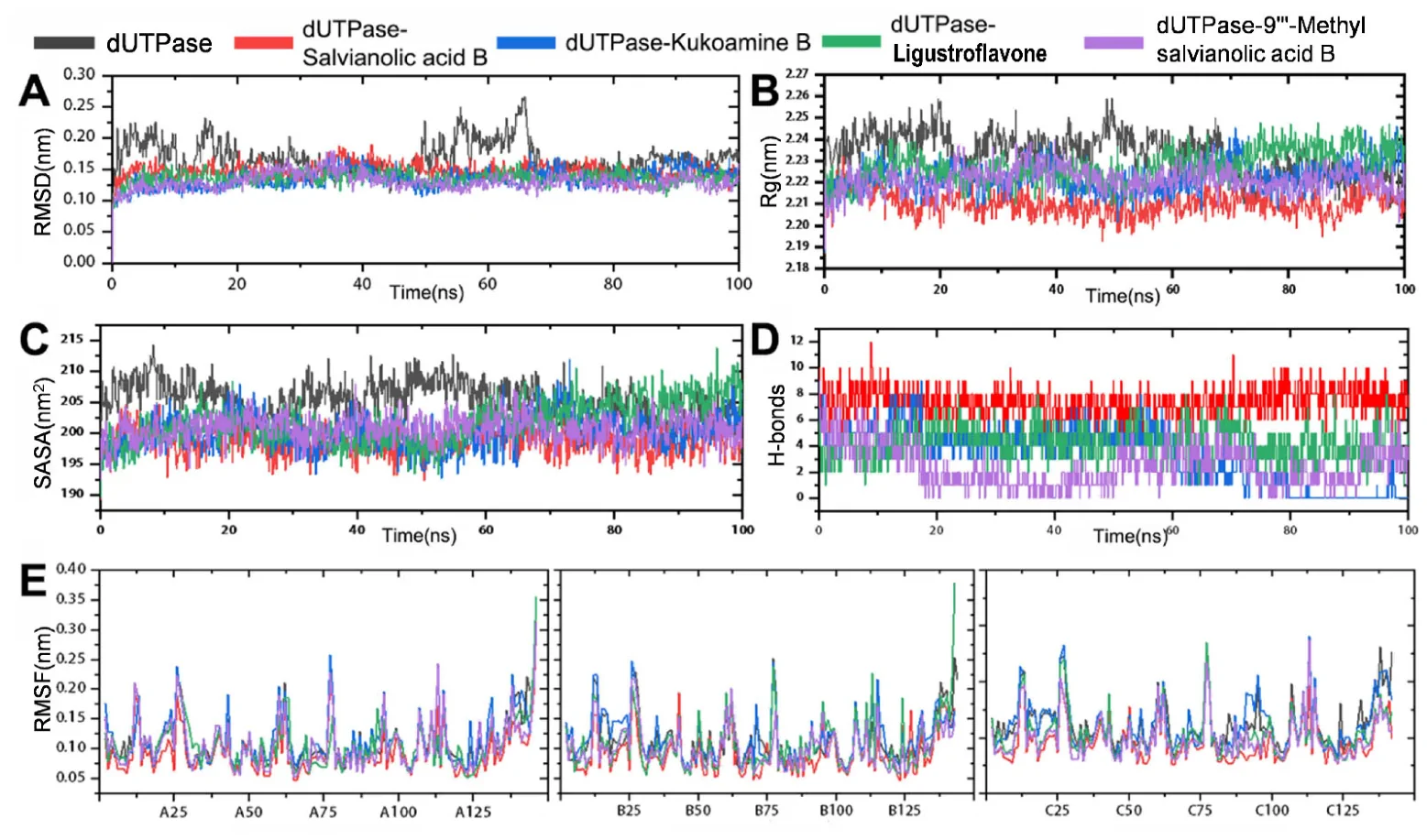
100 ns molecular dynamics (MD) simulations of apo-dUTPase and its complexes with four candidate inhibitors. (**A**) Root-mean-square deviation (RMSD) of the protein backbone atoms during the 100 ns simulation, (**B**) radius of gyration (Rg) analysis, (**C**) solvent accessible surface area (SASA) analysis, (**D**) number of protein-ligand hydrogen bonds, and (**E**) root-mean-square fluctuation (RMSF) of amino acid residues. Color legend: Gray, apo-state dUTPase; dark red, Salvianolic acid B-dUTPase complex; blue, Kukoamine B-dUTPase complex; green, Ligustroflavone-dUTPase complex; purple, 9‴-Methyl salvianolic acid B-dUTPase complex. Note: The residue numbering displayed on the RMSF axes corresponds primarily to the structurally ordered catalytic core mapped from the structural alignments. The flexible C-terminal segments (residues 151–165) were omitted from the production runs due to the intrinsic absence of experimental coordinates in the baseline crystals, thereby ensuring that the calculated fluctuation profiles reflect the stable geometric properties of the core active pocket without structural artifacts.

RMSD analysis (Figure 3A) indicated that all systems reached equilibrium after 5 ns, with stable trajectories maintained throughout the remainder of the simulation. The average RMSD of the apo-dUTPase was 0.266 nm. In contrast, the average RMSD values for the complexes with Salvianolic acid B, Kukoamine B, Ligustroflavone, and 9‴-Methyl salvianolic acid B were 0.190 nm, 0.179 nm, 0.164 nm, and 0.180 nm, respectively. These values were consistently lower than those of the apo-protein, suggesting that the protein structure maintains a certain conformational rigidity upon ligand binding. It should be noted, however, that such trajectory equilibration and geometric stability reflect dynamic structural accommodation within the binding pocket rather than serving as a direct or linear indicator of absolute functional inhibition capacity.

Radius of gyration (Rg) analysis (Figure 3B) showed that the Rg fluctuations of the complexes (<0.072 nm) were smaller than those of the apo-protein upon binding. Furthermore, during the mid-to-late stages of the simulation (>68 ns), the Rg values for the Salvianolic acid B and 9‴-Methyl salvianolic acid B complexes remained lower than that of the apo-dUTPase, suggesting a more compact structure. Solvent Accessible Surface Area (SASA) analysis (Figure 3C) was consistent with the Rg results; during the first 68 ns, all complexes exhibited lower SASA values than the apo-protein. The Salvianolic acid B complex displayed the lowest SASA, further confirming that its binding induced a more condensed protein conformation.

#### 3.2.2. Hydrogen Bond Interaction Analysis

Analysis of the number of protein-ligand hydrogen bonds (H-bonds) (Figure 3D) revealed that Salvianolic acid B formed the highest number of H-bonds with dUTPase (peaking at 12, with an average of ~7.4 per frame), while Kukoamine B formed the fewest (average ~2.4 per frame). Ligustroflavone and 9‴-Methyl salvianolic acid B exhibited average H-bond counts of 4.0 and 4.2 per frame, respectively. This result was basically consistent with the interaction intensity trends predicted by molecular docking.

#### 3.2.3. Residue Flexibility Analysis

RMSF analysis (Figure 3E) indicated that most residues showed no significant changes in RMSF values compared to the apo-state dUTPase, suggesting that inhibitor binding did not substantially alter the overall protein flexibility. Notably, while the initial trimeric simulation model spanned residues 2–149 for Chains A, B, and C as defined in Section 2.2, the residue indexing plotted along the horizontal axes in the fluctuation profiles was adaptively trimmed (spanning up to residue 146 for Chain A, and 143 for Chains B and C, respectively). This standard structural alignment protocol was implemented to avoid potential signal artifacts and geometric distortion originating from the flexible, unconstrained terminal tails during dynamic trajectories.

Regarding the functionally critical C-terminal flexible arm segment (residues 151–165), it was intrinsically unresolved in the baseline 6KY9 crystallographic coordinates. To ensure a high-contrast focus on the rigid catalytic core domain and avoid artificial thermal noise originating from the unconstrained terminal segments during dynamic trajectories, these flexible residues were omitted from the 100 ns production runs.

**Figure 4:**
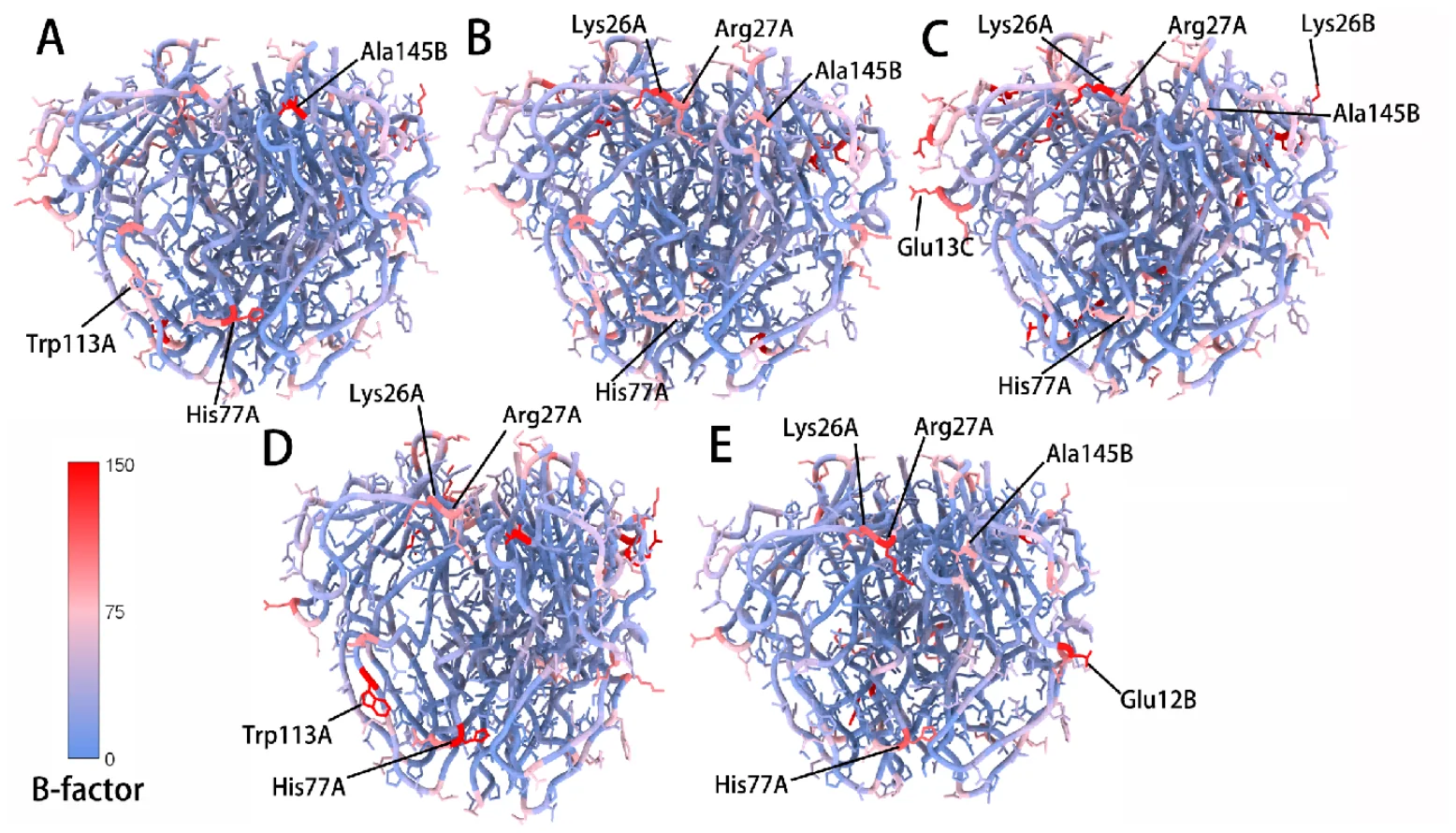
Residue flexibility distribution of apo-ASFV dUTPase and its complexes with four candidate inhibitors (B-factor mapping). (**A**) Apo-ASFV dUTPase, (**B**) Salvianolic acid B-ASFV dUTPase complex, (**C**) Kukoamine B-ASFV dUTPase complex, (**D**) Ligustroflavone-ASFV dUTPase complex, and (**E**) 9‴-Methyl salvianolic acid B-ASFV dUTPase complex. Protein structures are colored according to the B-factor gradient, ranging from blue (low B-factor/low RMSF, rigid) to red (high B-factor/high RMSF, flexible). B-factor values are converted from residue RMSF data.

Due to this adaptive terminal trimming, the residue numbering presented in Figure 3E focuses strictly on this well-ordered catalytic core domain. B-factor profiles derived from these stable core RMSF values further elucidated local flexibility changes (Figure 4). Lys26A and Arg27A, located around the active pocket, exhibited high flexibility across all systems. Notably, the binding of Ligustroflavone and Kukoamine B reduced the flexibility of His77A (Figure 4C,D), while Ligustroflavone simultaneously increased the flexibility of Trp113A (Figure 4D). Furthermore, the binding of Salvianolic acid B, Kukoamine B, and 9‴-Methyl salvianolic acid B decreased the flexibility of Ala145B (Figure 4B,C,E), suggesting these residues may play specific roles in inhibitor recognition.

#### 3.2.4. Dynamic Correlation Analysis

DCCM analysis was employed to characterize the motion correlations between the Cα atoms across the protein chains (Figure S1). To enhance the signal-to-noise ratio of the cross-subunit cooperative movements, the correlation matrices were constructed based on the rigidified catalytic core domain of each subunit. In strict alignment with the visualized matrix boundaries, the calculations spanned residues Ala2–Pro146 for Chain A, Ala2–Leu143 for Chain B, and Ala2–Ala143 for Chain C. In all systems, three distinct motion blocks corresponding to Chains A, B, and C were clearly observed. Compared to the apo-dUTPase, the binding of Kukoamine B and Salvianolic acid B significantly enhanced the correlative motions between residues of Chain A and Chain B (with both positive and negative correlations being strengthened). In contrast, the binding of Ligustroflavone and 9‴-Methyl salvianolic acid B primarily enhanced the positive correlations between Chain A and Chain B. These results indicate that different inhibitors exert varying effects on the internal dynamic network of the protein.

#### 3.2.5. Free Energy Landscape (FEL) Analysis

Free energy landscape (FEL) analysis based on principal component analysis (PCA) revealed that all complexes exhibited more centralized and narrower energy basins compared to the apo-dUTPase (Figure S2), suggesting a reduction in conformational entropy upon inhibitor binding. Among the candidates, the Salvianolic acid B complex displayed the most concentrated energy basin with the fewest intermediate states, indicating the highest conformational stability (Figure S2B). The energy basins for the Kukoamine B and 9‴-Methyl salvianolic acid B complexes were characterized by an elongated distribution along the Rg axis (Figure S2C,E), implying the existence of multiple conformational substates with similar energy levels. In contrast, the apo-dUTPase occupied a broader conformational space, with its energy basins diagonally dispersed along the RMSD and Rg axes (Figure S2A), reflecting its inherent conformational heterogeneity.

### 3.3. Binding Free Energy Calculation and Decomposition

The binding free energies of the four compounds with dUTPase were calculated using MM/PBSA and MM/GBSA methods, with the results summarized in Table 1 and Table 2. MM/PBSA calculations revealed that 9‴-Methyl salvianolic acid B exhibited the lowest binding free energy (−41.4 kcal/mol), indicating the strongest theoretical binding affinity, followed by Ligustroflavone (−35.9 kcal/mol) and Salvianolic acid B (−27.5 kcal/mol). Energy decomposition analysis identified van der Waals interactions (ΔEvdw) as the primary driving force for the binding of 9‴-Methyl salvianolic acid B and Salvianolic acid B, whereas electrostatic interactions (ΔEelec) contributed more significantly to the binding of Ligustroflavone.

Notably, the MM/PBSA binding free energy for Kukoamine B was positive (+13.5 kcal/mol), suggesting that its binding to dUTPase is thermodynamically unfavorable. This result contradicts its high docking score (−12.02 kcal/mol) obtained during virtual screening. Such a discrepancy may stem from the inherent differences in how docking scoring functions and free energy calculation methods account for solvation effects and entropic contributions, suggesting that Kukoamine B may not be an effective competitive inhibitor.

**Figure 5:**
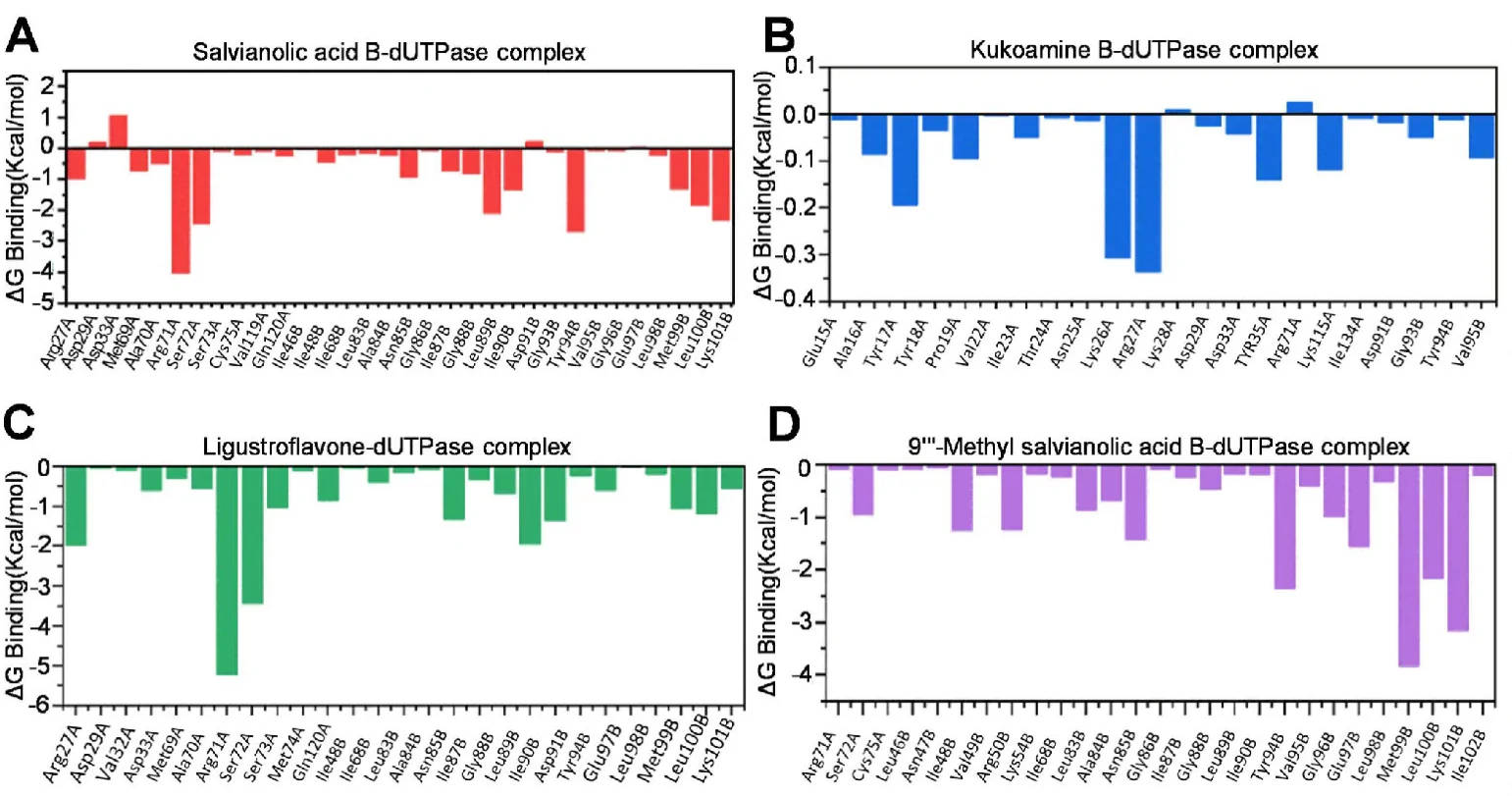
Per-residue binding free energy decomposition of ASFV dUTPase–inhibitor complexes based on MM/PBSA calculations. (**A**) Salvianolic acid B–ASFV dUTPase complex, (**B**) Kukoamine B–ASFV dUTPase complex, (**C**) Ligustroflavone–ASFV dUTPase complex, (**D**) 9‴-Methyl salvianolic acid B–ASFV dUTPase complex. The bars represent the energy contribution of key amino acid residues to the total binding free energy (ΔG Binding). Negative values indicate residues favorable for binding, while positive values indicate unfavorable ones.

Per-residue energy decomposition identified the key residues contributing to inhibitor binding (Figure 5), all of which are located within the active pocket of dUTPase. The binding of Salvianolic acid B was primarily driven by residues Arg71 and Ser72 of Chain A; and Leu89, Tyr94, Leu100, and Lys101 of Chain B. For Ligustroflavone, the binding involved Arg27, Arg71, and Ser72 of Chain A, alongside Asn85, Leu89, and Ile90 of Chain B. The contributing residues for 9‴-Methyl salvianolic acid B were more extensive, encompassing Leu89, Tyr94, and Ser73 of Chain A; Met99, Leu100, and Lys101 of Chain B; and Tyr94 of Chain C, which is consistent with its superior binding affinity.

### 3.4. Expression and Purification of Recombinant ASFV dUTPase

**Figure 6:**
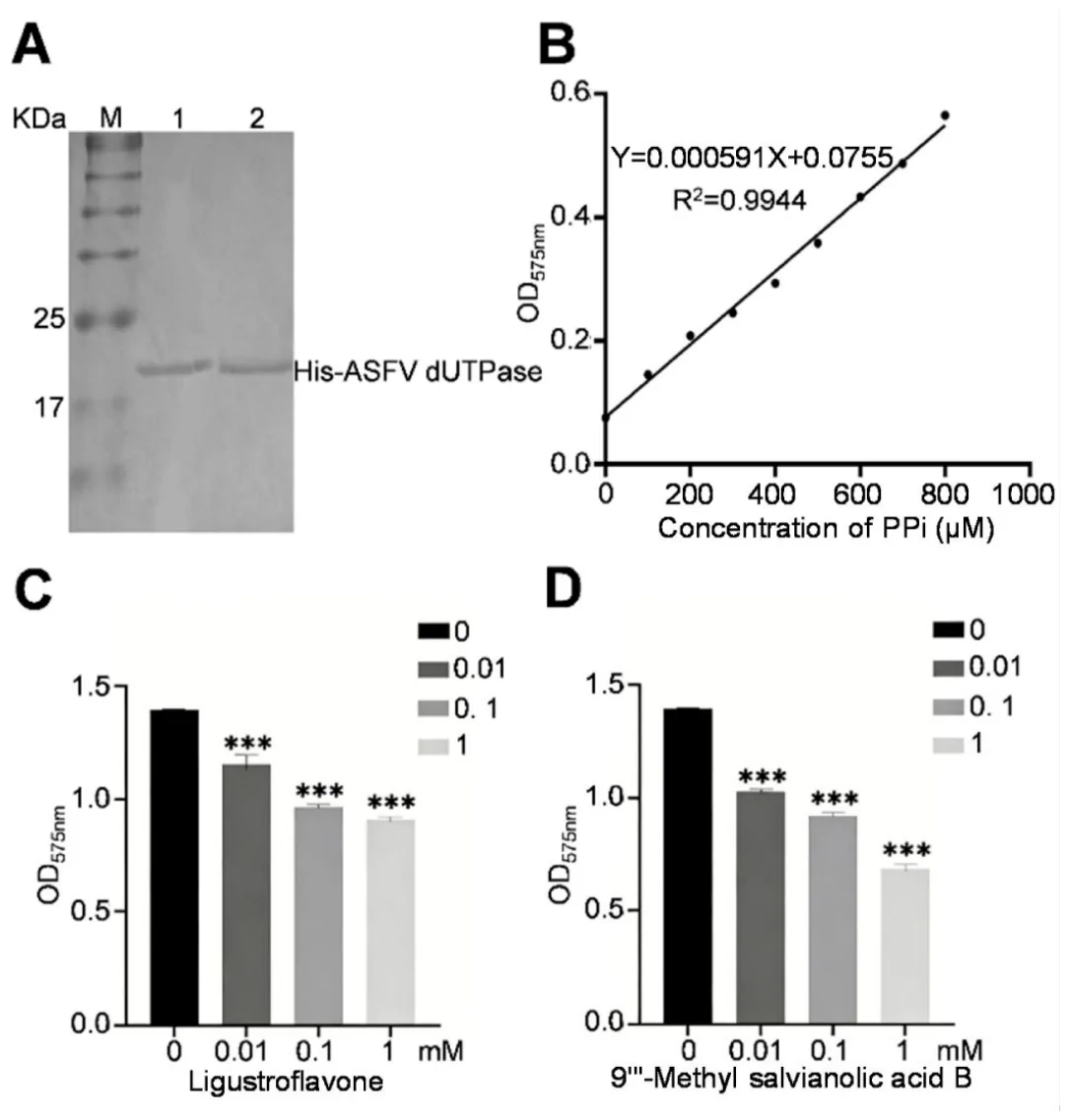
Purification, standard curve establishment, and enzymatic inhibition evaluation of recombinant ASFV dUTPase. (**A**) SDS-PAGE purification result of recombinant pET28a-His-ASFV dUTPase protein. Lane M: Protein molecular weight marker; Lane 1 and 2: Purified target protein. (**B**) Standard curve of pyrophosphate (PPi) measured at 575 nm (axes presented with equivalent mappings between concentration and absolute amounts). (**C**) Inhibitory effects of different concentrations of Ligustroflavone on ASFV dUTPase activity. (**D**) Inhibitory effects of different concentrations of 9‴-Methyl salvianolic acid B on ASFV dUTPase activity. Note: Data are presented as mean ± SD (n = 3). *** indicates a highly significant difference (*p* < 0.001) compared with the control group. The absorbance values for all compound-treated groups and the vehicle control group were systematically baseline-corrected by subtracting the optical density of their respective parallel compound-only blanks to eliminate any photometric interference from the native chromophoric skeletons of the natural products. Note: The high-absorbance baseline of the inhibitor-free control groups in the bar plots was quantitatively corrected using corresponding dilution scaling factors during data regression to avoid potential photometric saturation artifacts.

To perform in vitro enzymatic assays, the recombinant ASFV dUTPase protein was successfully expressed and purified. SDS-PAGE analysis (Figure 6A) revealed a single prominent band at approximately 19 kDa, consistent with the predicted molecular weight of the His-dUTPase fusion protein, indicating that a high-purity target protein was obtained. The concentration of the purified protein was determined to be 2.2 mg/mL using the BCA assay.

### 3.5. Verification of Enzymatic Inhibition Assays

A phosphomolybdate colorimetric calibration curve was initially established for the quantitative profiling of pyrophosphate (PPi). As demonstrated in Figure 6B, a linear relationship was achieved within the 0–800 μM concentration range (R^2^ = 0.9944, with the regression equation defined as: Y = 0.000591X + 0.0755, where X represents the molar concentration of PPi). Under these verified conditions, the specific activity of the purified recombinant dUTPase was determined to be 33.31 µmol·min^−1^·mg^−1^, calculated based on the linear velocity responses across balanced enzyme amounts ranging from 4.4 to 22.2 μg (Table 3). To capture high-contrast optical windows and clear colorimetric differentiation in the subsequent endpoint screening, a fixed loading of 2 μL purified enzyme solution was systematically implemented for the compound treatment assays (Table 4 and Table 5).

To evaluate the inhibitory efficacy of the four candidate compounds against dUTPase, biochemical assays were systematically executed across concentration gradients of 0.01, 0.1, and 1.0 mM (Table 4 and Table 5). The experimental tracking revealed that both 9‴-Methyl salvianolic acid B and Ligustroflavone exerted significant, dose-dependent inhibitory behaviors against dUTPase activity (Figure 6C,D). At a low concentration of 0.01 mM, 9‴-Methyl salvianolic acid B and Ligustroflavone achieved inhibition rates of 25.7% and 20.3%, respectively; upon escalating the treatment dosage to 1.0 mM, their inhibitory efficiencies advanced to 51.5% and 35.0%, parallel to prominent relative attenuations in the recorded OD_575_ inputs. The half-maximal inhibitory concentrations (IC_50_) were determined via non-linear regression profiles to be 0.92 mM for 9‴-Methyl salvianolic acid B and 1.87 mM for Ligustroflavone. Notably, although the absolute empirical IC_50_ values reside in the millimolar (mM) range, they exhibit qualitative trend consistency with the pharmacological rankings derived from the computational simulations. Specifically, 9‴-Methyl salvianolic acid B, which possesses a more favorable theoretical binding free energy than Ligustroflavone (−41.4 kcal/mol vs. −35.9 kcal/mol), mirrors this potency ranking in vitro by showing a lower IC_50_ readout. Conversely, Kukoamine B, which was predicted to be thermodynamically unfavorable by MM/PBSA (+13.5 kcal/mol), displayed no noticeable inhibitory activity across the tested concentrations, suggesting that computational free energy profiling provides relative prioritization in early-stage compound screening.

In excellent alignment with the theoretical modeling, Salvianolic acid B exhibited a weak, marginal inhibitory trend at an elevated concentration of 1.0 mM (inhibition rate of 22.8%), trailing behind its mono-esterified derivative 9‴-Methyl salvianolic acid B. Conversely, Kukoamine B failed to induce any observable enzymatic suppression across all evaluated concentrations (inhibition rate < 5%). This absence of biological phenotype strongly synchronized with the positive thermodynamic free energy (+13.5 kcal/mol) predicted by MM/PBSA calculations, further confirming from a wet-lab stance that Kukoamine B does not act as an effective functional inhibitor against ASFV dUTPase.

## 4. Discussion

In this study, a combined strategy of virtual screening, molecular dynamics (MD) simulations, and in vitro enzymatic assays was employed to successfully identify two lead compounds targeting ASFV dUTPase from a TCM library: 9‴-Methyl salvianolic acid B and Ligustroflavone. The value of ASFV dUTPase as an antiviral target lies in its essentiality for viral replication and the extremely low endogenous levels of dUTPase in the primary target cells (quiescent macrophages), which confers high potential selectivity to inhibitors targeting this enzyme [4,32]. However, given that functional genomics has demonstrated the non-essentiality of this enzyme for both in vitro replication and in vivo virulence in the highly virulent Georgia 2008/1 strain [5,6], its therapeutic relevance exhibits a distinct strain-dependent boundary. Thus, utilizing dUTPase for lead discovery in this work primarily leverages its highly conserved conformation as a structural probe to enrich the potential chemical space. While functional genomics verified that the E165R gene is non-essential for ASFV replication and virulence under controlled laboratory conditions [6], evaluating its potential as a therapeutic target could still be relevant within the context of wild-type field exposures. Under physiological stress or within specific resting host tissue niches where endogenous porcine dUTPase activity is limited, the viral enzyme may function as a supplemental safeguard to maintain genomic replication fidelity and mitigate dUTP misincorporation. Furthermore, characterized natural product scaffolds directed against this conserved active pocket could serve as preliminary structural starting points for future multi-target combination strategies aligned with other replication-associated enzymes, rather than being proposed as standalone monotherapeutic agents.

MD simulations and binding free energy calculations revealed that 9‴-Methyl salvianolic acid B exhibited the strongest binding affinity (−41.4 kcal/mol), primarily driven by van der Waals interactions. Per-residue energy decomposition indicated that this compound forms a stable interaction network with multiple residues, including Leu89(A), Tyr94(A), and Ser73(A); and Met99(B), Leu100(B), and Lys101(B), which is highly consistent with the hydrogen-bonding patterns predicted by molecular docking. Ligustroflavone showed a binding free energy of −35.9 kcal/mol, predominantly supported by electrostatic contributions involving residues such as Arg27(A), Arg71(A), and Ser72(A); and Asn85(B), Leu89(B), and Ile90(B). Notably, although Kukoamine B yielded a high docking score (−12.02 kcal/mol) in virtual screening, its MM/PBSA binding free energy was positive (+13.5 kcal/mol), suggesting that its binding to dUTPase is thermodynamically unfavorable. This discrepancy reflects the distinct thermodynamic division of labor between static docking scoring functions and free energy calculation methodologies in handling solvation effects and entropic contributions; this implies that following the initial screening phase, conventional static docking cannot fully encapsulate the dynamic evolution of ligands and the receptor within solution environments, thereby necessitating the complementary integration of binding free energy calculations for a multi-scale comprehensive evaluation [22]. These core thermodynamically contributing residues, identified via dynamic trajectory and energy decomposition, exhibit a rigorous biological correlation with the classically conserved active pocket of trimeric dUTPase. Comparative crystallography reveals that although the viral enzyme structurally mimics its porcine host counterpart in its macroscopic assembly as a symmetric homo-trimer, it distinctly constructs a novel, non-classical, two-subunit active site configuration at the microscopic level, dictated by the unique spatial orientation of its C-terminal β-strand following motif IV [3]. Within this distinct spatial boundary, each catalytic cleft is configured by the interfacial cooperation of two adjacent subunits and enclosed by the fold-back mechanism of the C-terminal flexible arm from its constituent interface [3] (Figure S3). Across the 100 ns trajectory, the two TCM monomers identified in this work (9‴-Methyl salvianolic acid B and Ligustroflavone) were stably accommodated within this two-subunit interface. Specifically, the positively charged basic amino acids, such as Arg27(A) and Arg71(A), are located within the functional zone responsible for anchoring the substrate phosphate groups, and their electrostatic interaction energy fluctuations during simulations are correlated with catalytic stability; concurrently, the critical residues at this adjacent inter-subunit boundary, including Tyr94(A), Met99(B), and Lys101(B), cooperatively form the uracil-recognition pocket determining substrate specificity. The dynamic simulation data acquired in this work are consistent with the steric occupancy and thermodynamic locking mode of this non-classical pocket: The compounds formed π-π aromatic stacking with the side chain of Tyr94(A) while simultaneously mediating hydrophobic interactions with Met99(B). This induced-fit engagement may sterically obstruct the entrance pathway of the substrate into the catalytic center, thereby providing a structural explanation for their observed in vitro inhibitory activities. Furthermore, the intrinsic conformational flexibility of the target protein may also influence the stable binding of inhibitors. Previous crystallographic studies [3] have demonstrated that the substrate-binding pocket of ASFV dUTPase is exceptionally spacious due to its unique non-classical two-subunit architecture, with a cavity volume approximately three times that of its porcine host counterpart. This topological feature suggests that the TCM-derived small-molecule monomers identified in this study tend to bind deeply within the internal core region of the cleft. Similarly, although the dynamic lid-like flipping and closure mechanisms of the unresolved C-terminal segment (residues 151–165) were not explicitly modeled in our core-domain trajectories to preserve convergence, previous structural studies indicate that this regulatory boundary plays an important role in substrate encapsulation. Structural comparison suggests that the identified TCM monomers tend to bind deeply within the internal region of the catalytic cleft, where the native flexible arm could potentially exert local hydrophobic shielding or auxiliary steric entrapment under physiological conditions.

In vitro enzymatic activity assays further demonstrated that 9‴-Methyl salvianolic acid B and Ligustroflavone exhibited preliminary inhibitory activity against ASFV dUTPase at millimolar (mM) concentrations, with IC_50_ values of 0.92 mM and 1.87 mM, respectively. Notably, such millimolar-level potency represents a typical initial hit profile for unmodified natural products in the early stages of virtual screening. In contrast, synthetic inhibitors such as TAS-114 [33] are the products of multiple rounds of rigorous medicinal chemistry optimization; therefore, the IC_50_ values identified in this study should be viewed as a valid starting point for subsequent structural optimization rather than the final endpoint of therapeutic lead maturity. Moreover, the inhibitory gradients of these compounds were generally consistent with the theoretical binding free energy trends (−41.4 kcal/mol for 9‴-Methyl salvianolic acid B vs. −35.9 kcal/mol for Ligustroflavone). This qualitative trend consistency is further supported by the observations that Salvianolic acid B displayed weak inhibitory activity at high concentrations (22.8% inhibition at 1.0 mM), whereas Kukoamine B showed no obvious inhibition, which matches its positive binding free energy prediction (+13.5 kcal/mol). These results collectively confirm the feasibility of deploying MM/PBSA to filter out thermodynamically unfavorable candidates and prioritize reliable natural product scaffolds. However, these computational thermodynamic metrics do not imply a direct linear correlation with biological efficacy on an absolute quantitative scale. From a biophysical perspective, MM/PBSA calculations based on molecular dynamics trajectories primarily quantify the theoretical thermodynamic equilibrium affinity (ΔG) under a specific closed conformation, carrying inherent methodological approximations in evaluating translational entropic penalties, local entropy costs, and conformational orientational changes. By contrast, the IC_50_ values derived from end-point biochemical assays are governed by complex kinetic inhibition behaviors in the solution microenvironment. For the structurally unoptimized natural product monomers in this study—characterized by bulky and highly flexible chemical frameworks (e.g., polyphenolic or iridoid glycoside scaffolds)—their penetration into the deep active cleft readily introduces multi-branch solvation dissociation kinetics and subtle steric mismatches, leading to deviations between theoretical thermodynamic scoring and empirical kinetic effects. Therefore, in the early-stage discovery of natural products, the core value of MM/PBSA free energy mapping resides in providing “gradient locking” of relative activities and scaffold prioritization, rather than serving as a rigid equivalent of absolute potency. Consequently, integrating binding free energy calculations into virtual screening workflows provides a more comprehensive thermodynamic basis for rational inhibitor prioritization.

Compared with existing studies, the compounds identified in this work demonstrated preliminary inhibitory activity against dUTPase. 9‴-Methyl salvianolic acid B, a derivative of Salvianolic acid B, was previously reported to possess anti-inflammatory and anti-platelet aggregation activities [34]. Through cross-screening, this study expands its potential utility to include the recognition of nucleotide metabolic enzymes. Ligustroflavone, recognized as a potential calcium-sensing receptor antagonist [35], has not been previously reported for applications targeting viral replication-related enzymes. In terms of established pharmacological profiles, natural monomers in antiviral drug discovery frequently present documented replication-inhibitory potentials and favorable low-cytotoxicity margins [36,37], providing a supportive biosafety background for exploring herbal scaffolds like salvianolic acid derivatives and Ligustroflavone. Their underlying mechanisms predominantly involve the modulation of cellular oxidative stress or the disruption of viral key replication enzymes. Currently, lead discovery efforts targeting enzymes essential for ASFV replication and nucleic acid metabolism have explored a variety of viral targets. In screening campaigns and structural biology investigations focusing on replication-essential enzymes, the small-molecule inhibitors triapine and cytarabine hydrochloride have been reported to effectively suppress the in vitro catalytic activities of key viral enzymes at micromolar concentrations by targeting ribonucleotide reductase and DNA polymerase, respectively [38]. Furthermore, m-AMSA precisely inhibits the catalytic activity of ASFV type II topoisomerase (pP1192R) via a distinct dual-functional mechanism [39]. Recently, Melendez et al. [40] successfully identified two potential natural product inhibitors derived from red algae (*Laurencia* species), namely thyrsenol A and 14-keto-dehydro-thyrsiferol, targeting ASFV dUTPase through an integrated computational screening workflow. This study provides robust methodological evidence for exploiting rational computational streams to discover active molecules from marine natural products that disrupt viral nucleotide metabolism enzymes. As systematically summarized in Table S1, a comparative statistical analysis of the interacting residues of ASFV dUTPase with our TCM small molecules and the red algal metabolites reveals high similarity at key interaction footprints, particularly for residues Asn85, Asp91, Tyr94, Met99, and Lys101. Conversely, the critical residue Glu97 shows a lower frequency of interaction among our prioritized TCM monomers. This structural divergence suggests that these small molecules may employ distinct fine-tuned binding modes with ASFV dUTPase, which warrants further experimental validation. Compared with the aforementioned red algal-derived polycyclic squalene-type derivative skeletons, 9‴-Methyl salvianolic acid B and Ligustroflavone identified from the TCM library in this study display entirely distinct terrestrial plant-derived polyphenolic (salvianolic acid derivatives) and iridoid glycosidic chemotypes. This not only significantly broadens the terrestrial plant-derived natural product chemical space for targeting this enzyme, but their dense induced-fit and spatial occupations within the non-classical inter-subunit uracil-recognition pocket (e.g., Tyr94(A) and Met99(B)) during 100 ns trajectories also form a beneficial structural complementarity with the micro-dynamic binding behaviors of the red algal monomers, thereby offering unique steric and topological references for subsequent structure-based rational modification of TCM components. These complementary screening efforts and structural insights collectively substantiate that anti-ASFV strategies targeting viral replication and nucleic acid metabolism-related enzymes are explicitly feasible, justifying our TCM database screening against dUTPase. In this context, although the inhibitory activities of the two identified TCM monomers during the initial screening phase remained at the millimolar (mM) level with relatively low potency, their spatial adaptation modes within this key enzyme regulating nucleotide pool balance offer a supplementary reference for uncovering anti-ASFV natural products. The relatively weak inhibitory potency of these natural small molecules during the initial screening stage may be attributed to steric hindrance and hydrophobic mismatch, which commonly arise from the larger molecular bulk and abundant polar groups that are characteristic of TCM-derived compounds. This characteristic inherently limits their baseline bioavailability to a certain extent. Furthermore, numerous natural product monomers targeting key replication enzymes of ASFV frequently exhibit similar weak-to-moderate activities in early in vitro screenings [36,37], suggesting that these natural scaffolds generally require subsequent rational structural modification. Future structure-activity relationship (SAR) investigations targeting this enzyme may focus on optimizing the ester-bond rigidity of 9‴-Methyl salvianolic acid B and introducing specific bioisosteric substitutions to the peripheral hydroxyl groups of Ligustroflavone, aiming to improve the cell membrane permeability and target binding affinity of the molecules while preserving critical steric hindrance within the core active pocket.

It should be noted that this study is explicitly positioned at the early stage of anti-ASFV drug discovery, focusing on target validation and the preliminary screening of lead compounds. The integrated strategy of “virtual screening–molecular dynamics simulations–in vitro enzymatic validation” employed in this work enables effective evaluation of the inhibitory potential of various compounds against ASFV dUTPase, while completely circumventing the need for live virus manipulation. Consequently, this work establishes a highly secure and practical screening pathway for discovering anti-ASFV leads in routine laboratories that lack BSL-3 facilities. Although the primary inhibitory profiles of these monomers have been confirmed at the cell-free enzymatic level, their cross-strain efficacy and pharmacological applicability must be systematically validated in future joint investigations using live-virus infection models under a certified BSL-3 platform, especially considering potential host compensatory mechanisms or alternative metabolic bypasses in virulent variants [5,6].

Furthermore, virtual screening campaigns targeting viral key proteins frequently encounter the potential risk of false-positive outcomes due to a primary reliance on static docking. In the computational workflow constructed in this study, although Kukoamine B exhibited a favorable docking score, the subsequent integration of all-atom molecular dynamics simulations and MM/PBSA-based binding free energy calculations successfully identified and predicted this thermodynamically unfavorable molecule prior to wet-lab experimentation. This multi-scale complementary strategy enables the investigation of dynamic conformational adaptation and binding thermodynamic behavior between ligands and the receptor in an environment closely mimicking real solvents, thereby providing a reliable kinetic basis for inhibitor prioritization [15]. Consequently, such a hierarchical evaluation strategy not only provides an effective methodological reference for screening anti-ASFV leads safely in routine laboratories but also further supports the scientific validity of rational computational workflows when handling complex natural product screening.

Finally, several methodology-specific caveats in our computational workflow must be acknowledged. Although the 100 ns molecular dynamics simulations successfully provided valuable microstructural and dynamic insights into short-term protein-ligand interactions, a single 100 ns trajectory remains inherently limited and may not fully chart the macroscopic conformational landscapes or longer-term thermodynamic equilibrium over extended physiological scales. Short-term single runs might inherently overlook potential continuous structural transitions or dissociation events over broader temporal domains. Consequently, the dynamic stability observed herein should be interpreted as preliminary structural accommodation. Future lead optimization efforts will focus on deploying microsecond-scale extended simulations and multi-replicate runs to establish a more definitive correlation between long-term structural thermodynamics and biological inhibition.

In conclusion, this study does not aim to present a fully mature and potent inhibitor but rather to provide a novel molecular perspective for the development of anti-African swine fever natural products through the virtual mapping and wet-lab verification of a curated TCM compound library. The structural-affinity discrepancy of Kukoamine B and salvianolic acid derivatives in docking scores versus binding free energies underscores the scientific validity and indispensability of multi-scale computation in natural product evaluation. 9‴-Methyl salvianolic acid B and Ligustroflavone exhibited promising levels of in vitro inhibitory activity at the millimolar (mM) scale, suggesting that their natural scaffolds can serve as starting probes for subsequent structure-based chemical modifications, thereby providing a novel scientific reference for exploring traditional Chinese veterinary medicine in the prevention and control of ASF caused by the Georgia 2008/1 strain.

## 5. Conclusions

In this study, two lead compounds targeting ASFV dUTPase were successfully screened and validated from a curated TCM library by integrating virtual screening, molecular dynamics (MD) simulations, and in vitro enzymatic assays. Our findings demonstrate the effectiveness of the “ADME-filtered computational screening–dynamic evaluation–experimental validation” strategy in identifying antiviral components from TCM resources. The identification of 9‴-Methyl salvianolic acid B and Ligustroflavone provides promising lead scaffolds for the development of novel anti-ASFV drugs specifically evaluated against the Georgia 2008/1 strain. Future efforts should focus on structural modifications to enhance their inhibitory potency, followed by further validation of their antiviral efficacy in cellular and animal models to facilitate their potential translation into clinical applications.

## Figures and Tables

**Table 1:** Binding free energies (MM/PBSA) of four candidate inhibitors with ASFV dUTPase (kcal/mol).

MM/PBSA (kcal/mol)	ASFV dUTPase- Salvianolic Acid B	ASFV dUTPase- Kukoamine B	ASFV dUTPase- Ligustroflavone	ASFV dUTPase- 9‴-Methyl Salvianolic Acid B
∆Evdw	−43.36 ± 3.08	−3.70 ± 8.42	−52.32 ± 4.40	−46.21 ± 3.49
∆Eelec	−41.38 ± 6.38	5.07 ± 8.11	−16.63 ± 4.06	−17.89 ± 7.02
∆Gpol	48.75 ± 6.94	−3.77 ± 7.32	25.50 ± 3.51	23.29 ± 5.23
∆GGAS	−84.74 ± 7.12	1.37 ± 9.28	−68.96 ± 7.00	−64.10 ± 6.16
∆GSOLV	43.71 ± 6.85	−4.26 ± 7.39	20.34 ± 3.27	18.84 ± 5.15
−T∆S	7.12 ± 0.05	9.28 ± 0.06	7.00 ± 0.06	6.16 ± 0.06
∆G Binding	−27.50 ± 2.35	13.50 ± 6.95	−35.87 ± 4.89	−41.40 ± 2.80

**Table 2:** Binding free energies (MM/GBSA) of four candidate inhibitors with ASFV dUTPase (kcal/mol).

MM/GBSA (kcal/mol)	ASFV dUTPase- Salvianolic Acid B	ASFV dUTPase- Kukoamine B	ASFV dUTPase- Ligustroflavone	ASFV dUTPase- 9‴-Methyl Salvianolic Acid B
∆Evdw	−43.36 ± 3.08	−3.70 ± 8.42	−52.32 ± 4.4	−46.21 ± 3.49
∆Eelec	−42.38 ± 6.36	5.07 ± 8.11	−16.63 ± 4.06	−17.89 ± 7.02
∆Gpol	45.46 ± 5.92	−3.95 ± 7.39	22.48 ± 3.06	22.86 ± 4.80
∆Gnonpol	−7.06 ± 0.29	−0.52 ± 1.16	−6.41 ± 0.55	−6.01 ± 0.36
∆GGAS	−84.74 ± 7.12	1.37 ± 9.28	−68.96 ± 7.00	−64.10 ± 6.16
∆GSOLV	38.40 ± 5.87	−4.47 ± 7.60	16.08 ± 2.65	16.84 ± 4.52
−T∆S	7.12 ± 0.05	9.28 ± 0.06	7.00 ± 0.06	6.16 ± 0.06
∆G Binding	−32.80 ± 2.55	13.29 ± 7.40	−52.88 ± 5.17	−43.40 ± 2.97

**Table 3:** Absorbance values at 575 nm of different amounts of recombinant ASFV dUTPase.

Tube Number	ASFV dUTPase Usage/μg	OD_575_	PPi Concentration (nmol)
1	4.4	0.529	76.734
2	8.8	0.554	79.949
3	13.2	0.588	86.717
4	17.6	0.627	93.485
5	22.2	0.650	97.208

**Table 4:** Effect of Ligustroflavone on the activity of recombinant ASFV dUTPase.

Tube Number	Volume Added (μL)					OD_575_
	ASFV dUTPase (2.2 mg/mL)	dUTP (100 mM)	Ligustroflavone (Stock Conc., mM)			
			0.01	0.1	1	
1	0	0	0	0	0	0.087 ± 0.003
2	2	0	0	0	0	0.102 ± 0.002
3	0	2.5	0	0	0	0.094 ± 0.005
4	2	2.5	0	0	0	1.386 ± 0.007
5	2	2.5	1	0	0	1.104 ± 0.089
6	2	2.5	0	1	0	0.958 ± 0.020
7	2	2.5	0	0	1	0.901 ± 0.014

Note: A volume of 1 μL of the chemical solution was added. In a total reaction volume of 100 μL, the final working concentrations in the assay are 0.1, 1, and 10 μM, respectively.

**Table 5:** Effect of 9‴-Methyl salvianolic acid B on the activity of recombinant ASFV dUTPase.

Tube Number	Volume Added (μL)					OD_575_
	ASFV dUTPase (2.2 mg/mL)	dUTP (100 mM)	9‴-Methyl Salvianolic Acid B (Stock Conc., mM)			
			0.01	0.1	1	
1	0	0	0	0	0	0.087 ± 0.003
2	2	0	0	0	0	0.102 ± 0.002
3	0	2.5	0	0	0	0.094 ± 0.005
4	2	2.5	0	0	0	1.386 ± 0.007
5	2	2.5	1	0	0	1.028 ± 0.017
6	2	2.5	0	1	0	0.911 ± 0.023
7	2	2.5	0	0	1	0.673 ± 0.041

Note: A volume of 1 μL of the chemical solution was added. In a total reaction volume of 100 μL, the final working concentrations in the assay are 0.1, 1, and 10 μM, respectively.

## Data Availability

The data presented in this study are available within the article.

## Supplementary Materials

The following supporting information can be downloaded at: https://www.mdpi.com/article/10.3390/v18080809/s1, Figure S1: Dynamic cross-correlation matrix (DCCM) analysis of apo-ASFV dUTPase and its complexes with four candidate inhibitors; Figure S2: Free energy landscape (FEL) analysis of apo-ASFV dUTPase and its complexes with four candidate inhibitors; Figure S3: Structural comparison of ASFV dUTPase and swine host dUTPase; Table S1: Comparative mapping of key interacting residues of ASFV dUTPase with terrestrial plant-derived TCM monomers, red algal metabolites, and the natural substrate dUTP.

## Abbreviations

The following abbreviations are used in this manuscript:

ASF	African Swine Fever
ASFV	African Swine Fever Virus
dUTPase	Deoxyuridine Triphosphatase
TCM	Traditional Chinese Medicine
MD	Molecular Dynamics
MM/PBSA	Molecular Mechanics Poisson-Boltzmann Surface Area
MM/GBSA	Molecular Mechanics Generalized Born Surface Area
CADD	Computer-Aided Drug Design
PDB	Protein Data Bank
RMSD	Root Mean Square Deviation
RMSF	Root Mean Square Fluctuation
Rg	Radius of Gyration
SASA	Solvent Accessible Surface Area
FEL	Free Energy Landscape
PCA	Principal Component Analysis
DCCM	Dynamic Cross-Correlation Matrix
PPi	Pyrophosphate
OD	Optical Density
IC_50_	Half-Maximal Inhibitory Concentration
PME	Particle-Mesh-Ewald
LINCS	Linear Constraint Solver
NVT	Constant Number-Volume-Temperature Ensemble
NPT	Constant Number-Pressure-Temperature Ensemble
H-bond	Hydrogen Bond
PLIP	Protein-Ligand Interaction Profiler
ADME	Absorption, Distribution, Metabolism, and Excretion
OB	Oral Bioavailability
DL	Drug-Likeness
SAR	Structure-Activity Relationship
SDS-PAGE	Sodium Dodecyl Sulfate-Polyacrylamide Gel Electrophoresis
BCA	Bicinchoninic Acid
