# Supplementary material for: Target-Oriented Screening of Traditional Chinese Medicine Potential Inhibitors Against African Swine Fever Virus dUTPase and Preliminary Evaluation of Enzymatic Activity Effects
Source: Viruses. 2026 Jul 23;18(8):809. doi: 10.3390/v18080809 (PMC13517570; doi:10.3390/v18080809)
Supplement: Supplementary file 1 [file viruses-18-00809-s001.zip › viruses-4388036-supplementary.pdf]

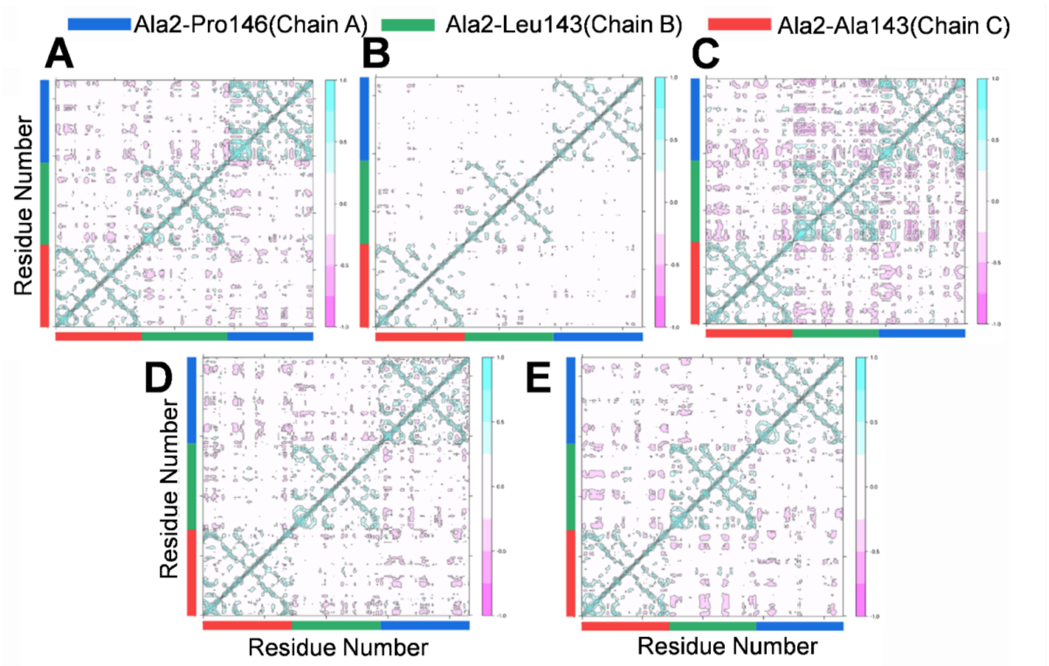

Figure S1. Dynamic cross-correlation matrix (DCCM) analysis of apo-ASFV dUTPase and its complexes with four candidate inhibitors. (**A**) Apo-ASFV dUTPase, (**B**) Salvianolic acid B-ASFV dUTPase complex, (**C**) Kukoamine B-ASFV dUTPase complex, (**D**) Ligustroflavone-ASFV dUTPase complex, (**E**) 9'''-Methyl salvianolic acid B-ASFV dUTPase complex. The matrices display correlated motions between Cα atoms of protein residues. Both axes represent residue number. The color gradient from blue through white to pink corresponds to negative (anti-correlated), uncorrelated, and positive (correlated) motions between residues, respectively.

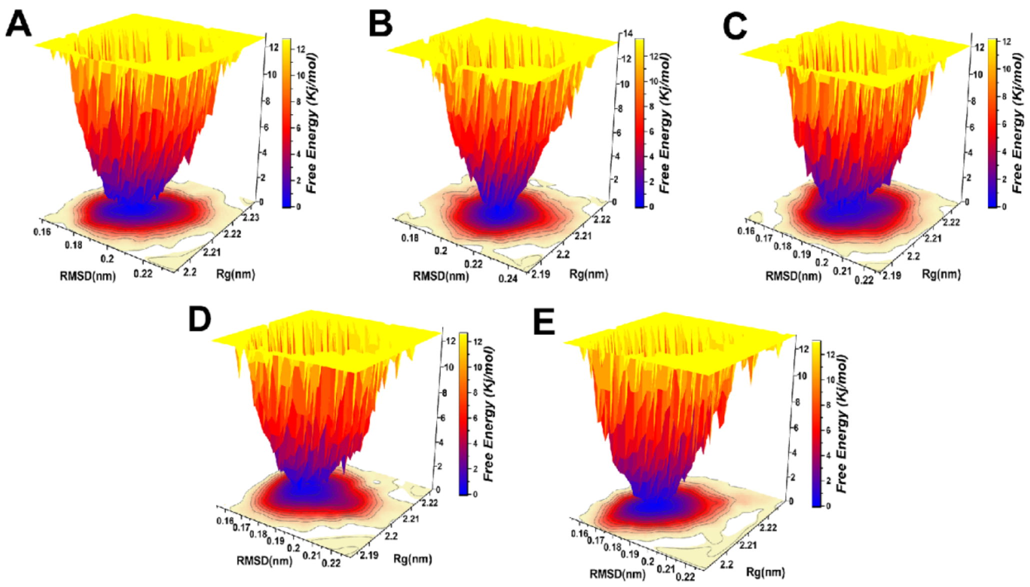

Figure S2. Free energy landscape (FEL) analysis of apo-ASFV dUTPase and its complexes with four candidate inhibitors. (**A**) Apo-ASFV dUTPase, (**B**) Salvianolic acid B-ASFV dUTPase complex, (**C**) Kukoamine B-ASFV dUTPase complex, (**D**) Ligustroflavone-ASFV dUTPase complex, (**E**) 9'''-Methyl salvianolic acid B-ASFV dUTPase complex. The free energy

landscapes are plotted using root-mean-square deviation (RMSD) and radius of gyration (Rg) of protein backbone atoms as coordinates. The color gradient from yellow through red to blue represents decreasing free energy, with blue regions corresponding to the lowest-energy stable conformational sub-states.

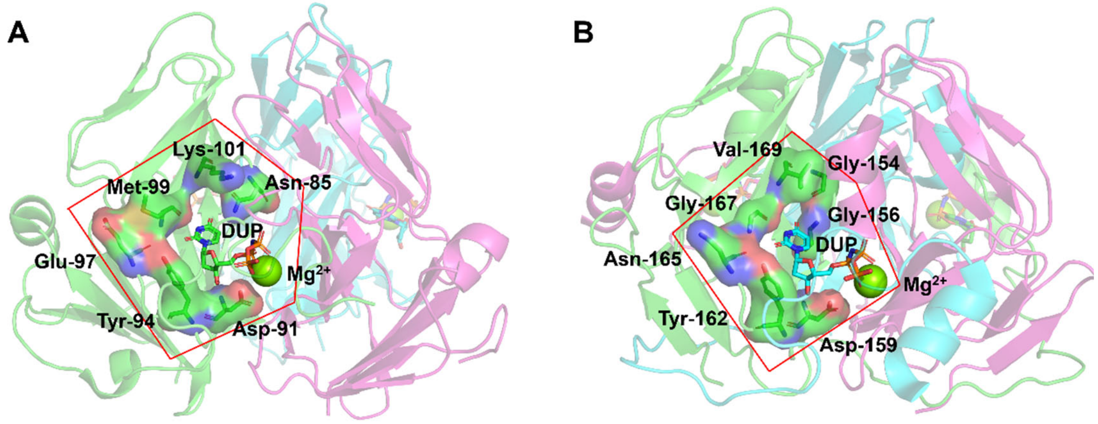

Figure S3. Structural comparison of ASFV dUTPase and swine host dUTPase. (**A**) Ribbon representation of the homotrimeric ASFV dUTPase. The substrate-capturing active site within Chain A (green) is highlighted using stick and semi-transparent surface models enclosed in a red solid polygon. Key active-site residues (Asn85, Asp91, Tyr94, Glu97, Met99, and Lys101), the ligand DUP, and the Mg^{2+} ion are explicitly labeled. (**B**) Ribbon representation of the homotrimeric swine (*Sus scrofa*) dUTPase. The corresponding substrate-capturing active site within Chain A (green) is highlighted using stick and semi-transparent surface models enclosed in a red solid polygon. Key active-site residues (Gly154, Gly156, Asp159, Tyr162, Asn165, Gly167, and Val169), the ligand DUP, and the Mg^{2+} ion are explicitly labeled. Individual subunits for both trimers are colored in green, pink, and cyan, respectively.

Table S1. Comparative mapping of key interacting residues of ASFV dUTPase with terrestrial plant-derived TCM monomers, red algal metabolites, and the natural substrate dUTP.

**Residues**	**dUTP**	**C0619**	**C1112**	**Salvianolic acid B**	**Kukoamine B**	**Ligustroflavone**	**9'''-Methyl salvianolic acid B**
Arg-27				O		O	O
Asp-29						O	
Asp-33					O		
Ile-48			O				
Val-49			O				
Ile-68	O	O	O				
Arg-71				O	O		O
Ser-72				O	O	O	
Ser-73				O			O

Leu-83	O	O	O				
Ala-84	O	O	O				
Asn-85	O	O	O	O	O		O
Gly-86		O					
Ile-87	O	O	O				
Gly-88	O	O	O				
Leu-89	O	O	O			O	
Ile-90	O	O	O	O			
Asp-91	O	O	O	O		O	O
Gly-93		O					
Tyr-94	O	O	O	O	O		O
Val-95		O					
Glu-97	O	O	O			O	
Leu-98	O	O	O				
Met-99	O	O	O	O		O	O
Leu-100	O	O	O				
Lys-101	O	O	O	O	O		O
Ile-102			O				
Lys-115						O	
Cys-117						O	
Gln-120						O	O

Note: "O" indicates the presence of molecular interactions between the residues and the corresponding ligands. The residue interaction coordinates for the natural substrate dUTP and the red algal metabolites (C0619 and C1112) are adapted and compiled from Melendez et al. (*Curr. Drug Discov. Technol.*, 2026, Online ahead of print, DOI: 10.2174/0115701638415372251124101006).
